# Competencies required for general practitioners/family physicians in urban areas versus non-urban areas: a preliminary study

**DOI:** 10.1186/s12875-018-0869-4

**Published:** 2018-11-29

**Authors:** Toshichika Mitsuyama, Daisuke Son, Masato Eto

**Affiliations:** Department of Medical Education Studies, International Research Center for Medical Education, Graduate School of Medicine, The University of Tokyo, 7-3-1 Hongo, Bunkyo-ku, Tokyo, 113-0033 Japan

**Keywords:** General practitioner, Family physician, Competency, Urban area, Non-urban/rural area

## Abstract

**Background:**

The medical practice of general practitioners/family physicians in urban areas differs from that in rural areas, accounting for the difference in specific competencies. However, variations in competencies in community healthcare required for general practitioners/family physicians in urban areas compared with those in rural areas have not yet been fully clarified. Thus, this study aimed to elucidate the competencies required for general practitioners/family physicians, especially in those characteristic to urban areas, and compare them with those in non-urban/rural areas.

**Methods:**

A qualitative study with individual interviews and qualitative data analysis was conducted. Participants were selected by purposive sampling, and 10 general practitioners/family physicians with clinical experience of ≥7 y after graduation and ≥ 1 y in both urban and non-urban (rural) areas in Japan were recruited. Additionally, semi-structured individual interviews in a private room around the workplace of the interviewee between September 2014 and September 2016 were conducted. For data collection, interview transcripts were analyzed according to the “Steps for Coding and Theorization” method, a sequential and thematic qualitative data analysis technique and data analysis since March 2018.

**Results:**

We interviewed 10 general practitioners/family physicians of Japan and extracted 10 themes as competencies characteristic to general practitioners/family physicians in urban areas. In addition to the known competencies on urban underserved care, we newly clarified the competencies of the ability to integrate divided care and ability to coordinate and collaborate with various medical care and welfare professionals in urban areas.

**Conclusion:**

This study was one of the few studies describing the characteristic competencies of urban general practitioners. In summary, a competency necessary for general practitioners in urban areas is to understand the urban context and provide contextual care suitable for urban areas. In the modern age, where urban population concentration is progressing and the interest in urban health is rising, our study will give certain suggestions for primary care education and practice necessary for urban areas.

## Background

### Primary care in urban and rural areas

Various socioeconomic factors, such as income, education level, access to medical services, and surrounding environment, have been established to affect the health of people living in a regional area [1, 2]. However, in urban and rural areas, socioeconomic factors comprise structurally different characteristics, and the effects of this status on health would differ between urban and rural areas [3–5]. Additionally, the provision of primary care is an effective means to enhance residents’ health outcomes while taking into account their various socioeconomic factors [6]. Therefore, the competencies required of general practitioner/family physicians (hereinafter general practitioners), which is one of the important players of primary care, should also have different characteristics depending on various socioeconomic factors in urban and rural areas [7, 8].

From the viewpoint of comparison between urban and rural areas, some aspects are already known about the provision of primary care and education of doctors responsible for primary care. For example, in the United States, The National Health Service Corps stated that it is important to make improvements so that residents have more accessibility to primary care by increasing the number of primary care physicians with community-responsive and culturally competent abilities not only in rural areas but also in urban areas. In rural areas, a relative shortage in health care resources and limited geographical access to medical institutions have been highlighted, and responsible for primary care play essential roles in providing comprehensive medical care [1]. On the contrary, in urban areas, medical resources are abundant, but most physicians are specialists, and those responsible for primary care are limited. Besides this skewed distribution of physicians among departments [9], care for the urban, underserved population, who cannot obtain adequate public services in terms of sociocultural and economic status, including minority race groups and groups with limited economic access to medical services because of income and health insurance disparities, has drawn attention [3, 6, 10]. In addition, the American Academy of Family Physicians (AAFP) also describes the necessary outline for urban general practitioner/family physicians programs as “Urban/Inner-City Training Program in Family Medicine” [11]. This guideline includes the need for doctors to receive training to provide care for the urban underserved and culturally effective community-responsive primary care. From these facts, the general practitioners in urban areas need to establish and obtain unique competencies different from those in rural areas.

However, limited studies have investigated the competencies of general practitioners in urban areas compared with those in non-urban (rural) areas especially from the educational perspective, and several available studies have only considered partial clinical aspects. For example, some studies include differences in disease profiles managed by family physicians in urban and non-urban areas in the United States [12], a comparison of hypertension management by general practitioners in urban and rural areas of Australia [13], a survey of general practitioners in urban areas of Canada on practice of minor procedures [14], and a comparison between urban and rural areas on quality improvement activities by primary care physicians in the United States [15].

### Japanese setting

In Japan, the Japanese Family Medicine Association (now changed to the Japan Primary Care Association) started the accreditation system of family physician specialist and primary care physician in 2006, and 673 family physicians have been certified by 2018 [16]. Then, while the Japanese Medical Specialty Board was established in 2014 as an independent third-party institution to guarantee the quality of training programs and medical specialists, the specialized training of community-responsive general practitioners has just begun [17]. Thus, in Japan, the name of accredited primary care physician changes according to age with the family physician and general practitioner, but we treat both as synonymous in this article. In contrast to the gradual development of primary care and home medicine in Japan, highly urbanized areas of Japan that have reached super-aged societies earliest among developed countries face several challenges on urban medical care and urban primary care, which other countries will face in the future. To illustrate what healthcare issues are faced by urban areas in Japan, how these issues are perceived by general practitioners responsible for the urban primary care and what skills they perceived are necessary to solve these issues are likely to facilitate the future provision of primary care and general practitioner training in urban areas around the world. Thus, this study aims to elucidate the competencies required for general practitioners/family physicians, especially in those characteristic to urban areas compared with non-urban/rural areas of Japan.

## Methods

### Research design

We conducted a qualitative study with 10 individual interviews separately. In parallel with data collection, interview transcripts were analyzed according to the “Steps for Coding and Theorization” method (SCAT), a sequential and thematic qualitative data analysis technique [18].

### Participants

We selected participants through purposive sampling between September 2014 and September 2016 and recruited Japanese general practitioners/family physicians with clinical experience ≥7 y after graduation and ≥ 1 year experience in both urban and non-urban (rural) areas in Japan. In this study, a general practitioner was defined as a family physician or a primary care physician certified by the Japan Primary Care Association.

Although there is no common global definition of urban areas, we applied the categories of “Major Metropolitan Areas” and “Metropolitan Areas” which is the major definition by the Statistics Bureau of Japan [19]. And all the remaining areas are defined “Non-urban/Rural”. These categories were also adopted in the United Nation’s report [20]. “Major Metropolitan Areas” consist of “central city(ies)” and “surrounding areas” (Shi, Machi and Mura) which have a high degree of economic and social integration [19]. In addition, we use another definition of urban areas by the Statistics Bureau of Japan which is based on a population density as supplemental information [21].

All participants had to have at least 7 y of postgraduate training to represent general practitioners who completed the initial training and advanced training, which usually takes 2 and 3–4 y, respectively. All participants in this study had to have clinical experience in both urban and non-urban (rural) areas because this study focused on the characteristic of urban areas compared with non-urban areas, and physicians with clinical experience in both regions were more likely to speak more specifically about the comparison between urban and rural clinical practices based on their own experience.

### Data collection

We conducted semi-structured interviews with each interviewee in a private room around their workplace in Japan between September 2014 and September 2016. First, we asked participants to participate in this study by e-mails, and those who agreed to do so were explained the purpose/procedure/ethical considerations of this study in detail and included in interviews. The interviews were semi-structured and performed individually by the primary investigator (TM). Each interview took approximately 60–100 min. Of note, all interviews were conducted in a private room to maintain privacy and recorded with the subjects’ permission to construct verbatim records.

A semi-structured interview primarily comprised the following questions: “What kind of patients are you treating now? In which area?” “What are the skills required to work in this area?” “Do you think there are any differences in skills required for general practitioners in urban versus rural areas? Why?” “Do you think some skills required for general practitioners in urban versus rural areas are the same? Why?”

### Data analysis

Data analysis was simultaneously conducted with data collection, the transcripts of the interviews were analyzed according to SCAT [18]. SCAT consists of several steps for data analysis. First, text was divided based on the verbatim records. Second, texts were coded with emerging themes. Thereafter, a story line was written by using the themes, and theoretical description was made from the story line [18, 22]. These processes were conducted on each transcript of the interviewees. Finally, an integrated story line and theoretical description were created by combining all the concepts. These processes were primarily performed by the main author (TM), and the codes and story lines were confirmed by each coauthor (DS, ME). And we returned each story line and theoretical description of each interviewee and requested confirmation as to whether they were in line with the intention of remark. As a result, there was no particular objection or correction request from the participants. We aimed to finish sampling and collecting data when we obtained no new themes and reached the theoretical sufficiency [18]. We finished all the process of data analysis in March 2018.

## Results

From September 2014 to September 2016, we conducted qualitative interview and analysis for 10 general practitioners of Japan. Because we could not obtain the data that explained new concepts on urban general practitioner’s competencies after the 10 interviews and analysis, we judged that theoretical sufficiency was reached and finished the sampling. The characteristics of the 10 general practitioners who participated in the interview are provided in Table 1. The median post graduate year of participants was 9.3y; six of the ten were female. Four present workplaces were urban clinics, three were urban hospitals, and three were rural clinics. All ten were experienced in both urban and non-urban (rural) areas. All the urban workplaces were Major Metropolitan Areas and the population density of all the urban cities were 5000 or more per square kilometer.

**Table 1:** Attributes of study participants

ID	Gender	PGY/ Age	Medical specialty	Present work place	Urban area of workplace (population number/density) Prefecture name, workplace and years	Rural area of workplace (population number/density) Prefecture name, workplace and years
1	Male	15/ 40	FP	rural CL	Central City (> 200,000/ > 5000) Hokkaido, Sapporo City, MC, 5 y	Municipal Village (~ 3000/ ~ 20) Hokkaido, CL, 11y
2	Female	7/ 31	FP	urban CL	Central City (> 900,000/ > 15,000) Tokyo, Setagaya Ward, CL, 6y	Municipal City (~ 60,000/ ~ 2000) Saitama, LH, 1y
3	Female	10/ 34	FP	rural CL	Central City (> 2,500,000/ > 10,000) Osaka, Osaka, CL, 2y	Municipal City (~ 40,000/ ~ 400) Chiba, CL, 6y
4	Female	7/ 34	FP	urban CL	Central City (> 1000,000/ > 15,000) Tokyo, Taito Ward, CL, 1y	Island (~ 1000/ ~ 100) Okinawa, CL, 6y
5	Male	8/ 32	FP	urban CL	Surrounding area (> 100,000/ > 10,000) Tokyo, Koganei City, 2y	Island (~ 1000/ ~ 50) Okinawa, CL, 6y
6	Female	10/ 34	FP	urban CL	Central City (> 1000,000/ > 10,000) Tokyo, Adachi Ward, CL, 4y	Municipal Town (~ 5000/ ~ 6000) Saitama, CL, 6y
7	Male	9/ 33	DPC	rural CL	Surrounding area (> 700,000/ > 7000) Osaka, Sakai City, MC, 5y	Island (~ 9000/ ~ 30) Kagoshima, CL, 4y
8	Male	10/ 34	FP	urban LH	Central City (> 500,000/ > 15,000) Tokyo, Suginami Ward, LH, 7y	Municipal City (~ 40,000/ ~ 400) Chiba, CL, 3y
9	Female	9/ 33	FP	urban LH	Central City (> 300,000/ > 15,000) Tokyo, Kita Ward, LH, 3y	Municipal City (~ 20,000/ ~ 70) Kochi, LH, 6y
10	Female	8/ 32	FP	urban LH	Central City (> 300,000/ > 15,000) Tokyo, Kita Ward, LH, 3y	Municipal City (~ 40,000/ ~ 500) Shizuoka, CL, 5y

*FP* certificated family physician, *DCP* diplomate in primary care, *MC* medical center, *LH* local hospital, *CL* clinic, *PGY* post graduate year; population density unit is per square kilometer

As we analyzed the interview data, it became clear that the competencies of general practitioners in urban areas compared with those in non-urban (rural) areas were greatly affected by differences in their working environment, such as available health services, and we perceived this to be an inseparable relationship. Therefore, we also broadly analyzed narratives on how the participating general practitioners perceived their work environment as additional themes. As shown in Table 2, these themes were organized into the following five groups: (1) competencies characteristic of general practitioners in urban areas; (2) contexts characteristic of general practitioners in urban areas; (3) competencies characteristic of general practitioners in non-urban areas; (4) contexts characteristic of general practitioners in non-urban areas; and (5) common competencies between both groups of general practitioners.

**Table 2:** Competencies and contexts of general practitioners in urban/non-urban areas and common competencies

Category	Concepts
Competency of general practitioners in urban areas	• Demonstration of comprehensive care ability depending on conditions • Integration of fragmented care in urban areas • Active involvement in patients who received fragmented care • Comprehensive care for minority group, a characteristic of each region of urban areas • Understanding various occupations/lifestyles in urban areas • Formation of agreements with patients with various values in urban areas • Judgment for appropriate hospital introduction according to the patient situation • Efforts for regional collaboration on emergency medicine issues in urban areas • Collaboration with various medical care and welfare personnel • Communication with nonresident family members
Context of general practitioners in urban areas	• Relatively narrow scope of biomedical care • Patients’ selective care-receiving behavior in urban areas • Segmented healthcare services in urban areas • Unclear responsibility regarding care • Confusion about what being a general practitioner in urban areas means • Diversity of socioeconomic regional characteristics in an urban area • Various occupations/lifestyles in urban areas • Relatively high healthcare needs in urban areas • Sense of difficulties in understanding different medical resources • Quality differences in medical care among physicians/hospitals in urban areas • Emergency medicine issues in urban areas • Difficulties in comprehensive local community care • Diversity of local medical care and welfare professionals • Lack of face-to-face relationships in medical care and welfare collaboration in urban areas • Lack of mutual help function around patients in urban areas • Long physical/psychological distance between workplaces and homes • Lack of visibility of families and affiliated communities
Competency of general practitioners in non-urban areas	• Broad biomedical care scope • Responsibility of doctors as limited medical resources • Judgment to make effective use of limited medical resources • Care collaborating with local communities • Ability to build appropriate human relationships with residents
Context of general practitioners in non-urban areas	• Clarity of responsibility of care • Ease of maintaining interpersonal continuity • Ease of acquiring identity as a family physician in non-urban areas • Regional differences in medical care-receiving behaviors in non-urban areas • Limited medical resources • A sense of understanding medical care skills of surrounding medical institutions and individual physicians • Face-to-face relationships in healthcare collaboration • Ease of grasping local communities • Face-to-face relationships in medical care and welfare cooperation • Physical/psychological proximity between workplaces and homes • High visibility of patient/family background
Common competency of general practitioners in urban/non-urban areas	• Biomedical care ability • Comprehensiveness of medical care according to place and situation of medicine • Medical care for patients with multiple diseases • Healthcare workers as support roles in each patient’s life • Necessity of decision-making based on patient background • Connection role in community healthcare • Division of labor/collaboration with subspecialists in hospitals • Creation of social resource networks for community care • Familiar advisors

Particularly, the following 10 themes were obtained as “competencies of general practitioners in urban areas”: (1) Demonstration of comprehensive care ability depending on conditions; (2) Integration of fragmented care in urban areas; (3) Active involvement in patients who received fragmented care; (4) Comprehensive care for minority group, a characteristic of each region of urban areas; (5) Understanding various occupations/lifestyles in urban areas; (6) Formation of agreements with patients with various values in urban areas; (7) Judgment for appropriate hospital introduction according to the patient situation; (8) regional collaboration efforts for emergency medical issues in urban areas; (9) Collaboration with various medical care and welfare personnel; and (10) Communication with nonresident family members.

Among the competencies and contexts characteristic of urban general practitioners, particular core themes were shown below.

### Demonstration of comprehensive care ability depending on conditions

One of the common competencies of both urban and non-urban general practitioners is that they can provide a wide range of biomedical care and integrated care to patients with multiple diseases. General practitioners said that in non-urban areas the surrounding medical resources are limited and patients tend to concentrate on their clinics, so that it is easy to demonstrate a broad scope of biomedical care and to provide integrated care. By contrast, they reported that in urban areas the scope of biomedical care is relatively narrow because of patient behavior of selectively receiving care and segmented healthcare services.

Representative texts for each theme were shown below. And following the texts, IDs, Gender and post graduate year (PGY) of the participant are shown in parentheses that correspond to Table 1.

*“Patient goes to a lot of hospitals, for example, to an orthopedics for osteoporosis, to this clinic for hypertension, and go to a otolaryngology for colds.” (ID5, male, 8PGY)*

*“I can do joint injections, but I am not an orthopedic specialist and some patients do not want to receive joint injections here…. I think it might be good to divide labor whenever possible.” (ID5, male, 8PGY)*

As a result, general practitioners in urban areas must have competencies to [Demonstration of comprehensive care ability depending on conditions], which provide flexible medical service according to the patient’s individualized needs and medical care provided by surrounding specialized medical institutions.

*“There are many patients I cannot refer (to another physician)… so whoever comes, you need to have some degree of curiosity toward patients. It would not be possible for me to say that this is not my expertize…” (ID6, female, 10PGY)*

### Active involvement in patients who received fragmented care

Moreover, because in urban areas patients who have multiple diseases had consultation with multiple specialists, the responsibility of care tends to be obscured.

*“I guess there are a lot of people visiting multiple hospitals. If something wrong with them, they may call an ambulance. At that time, there is no one who takes final responsibility for the health of that person. In other words, I feel that responsibility is quite unclear.” (ID5, male, 8PGY)*

It is important that the general practitioner actively plays a role and is responsible for the overall picture of the patient.

*“Primary care doctors responsible for the overall picture are certainly necessary. Especially for the elderly…if they are treated for a health problem outside the specialty of the current attending physician or for a health problem that has no medical name, no one is responsible and care managers often have difficulties.” (ID 8, male, 10 PGY)*

### Integration of divided care in urban areas

Furthermore, having the ability to organize patient information that tends to be divided for patients who are visiting multiple hospitals and to integrate disease management because of difficulties in visiting due to aging or disability is necessary.

*“There are various hospitals, and a patient may visit, for example, five hospitals per week for different treatments, but he is tired and does not want to go anymore to a hospital. For such patients, I kindly suggest ‘if you are visiting too many hospitals, you can cut about two of them and receive the treatments here.’” (ID5, male, 8PGY)*

### Comprehensive care for minority group, a characteristic of each region of urban areas

General practitioners who participated in the study thought that the competencies of general practitioners in urban/non-urban areas are to take care of individual patients’ lives according to the patient background. Compared with non-urban (rural) areas, urban areas are more diversified in terms of socioeconomic status and occupations/lifestyles. They thought that understanding diversity and providing flexible care are important and roles in urban areas.

*“… I think that it is certainly necessary to have the ability to respond flexibly to what people are seeking.” (ID2, female, PGY7)*

In particular, general practitioners thought that providing foreign residents, who are a minority group, with medical needs specific to each culture is one of the characteristic competencies in urban areas.

*“There are quite a few people who raise children in an isolated manner because the father, mother, and grandparents are far away or because they are foreigners and are not fluent in Japanese. Many moms cannot make “Mama-tomo (mother friends),” so it may become necessary to spend some time talking with such people during an infant health checkup or the like.” (ID6, female, 10PGY)*

### Judgment for appropriate hospital introduction according to the patient situation

Commonly, in both urban and rural areas, general practitioners have a wide range of medical treatment abilities and the ability to properly introduce them to organ specialists. In non-urban areas, it is possible to build “face-to-face” relationships in medical cooperation and to grasp the medical care capacity for each medical institution because medical resources are limited. One competency required for general practitioners in non-urban areas is [the judgment to make effective use of limited medical resources]. By contrast, there are various medical resources in urban areas, and general practitioners feel the disparity in the quality of medical care by hospitals and have difficulties in understanding the wide range of medical resources.

*“There are too many hospitals, and I do not know where (hospital) is the best (for some disease). Then, I feel that I cannot build a face-to-face relationship.” (ID8, male, PGY10)*

The doctor feels that more complicated judgment ability is required when introduction to a specialist becomes necessary. It is the ability to introduce patients to a specialist by taking into consideration the patient’s biomedical situation and the characteristics of surrounding medical institutions from among several options.

*“There are plenty of hospitals in the area, so I can refer a patient anywhere, but I may have more trouble if I introduce a patient to the wrong hospital. Honestly, if I had more face-to-face relationships with other medical personnel, I would not question as much whether more specialized physicians, or actually a certain physician, or hospital would be better.” (ID6, female, 10PGY)*

### Collaboration with various medical care and welfare personnel

In addition, as a role of general practitioners, emphasis is placed not only on the medical cooperation mentioned above but also on the collaboration for interprofessional work in local communities and health promotion with residents. In non-urban areas, providing care in cooperation with local communities is easily performed because of the ease of understanding among local communities and face-to-face relationships with medical care and welfare groups [23, 24]. By contrast, general practitioners in urban areas are required to collaborate with various medical care and welfare personnel, as comprehensive community care is difficult because of the diversity of medical care and welfare personnel and the lack of face-to-face relationships.

*“I think it may be even harder to demonstrate leadership in community collaboration if I go to an urban area… I think cooperation is unmanageable or hard to achieve… I think it is more difficult in urban areas. Even with the same effort, a higher level (of management) might be required.” (ID1, male, 15PGY)*

## Discussion

In this study, we focused on the competencies required for general practitioners working in urban area and compared such competencies with those in non-urban areas. Moreover, we tried to clarify them by interviewing 10 general practitioners working in both regions and analyzing their interview transcripts qualitatively. We found 10 competencies characteristic of urban general practitioners and some themes about the contexts that affect competencies. As a characteristic context in urban areas, urban areas are composed of people with diverse socioeconomic conditions and medical needs compared to non-urban areas. Further, some situations call for selectively using various medical services, which even care providers cannot grasp. In such contexts, competencies required for general practitioners in urban areas included comprehensive medical care ability according to various situations, the ability to respond to diverse patient backgrounds/values, the ability to integrate divided care, and the ability to cooperate with various medical care and welfare personnel.

### Contextual care

Our results indicate that the competency characteristic of urban comprehensive medical doctors is inseparable from each other based on the context of urban areas. This is related to the theoretical framework called contextual care in primary care. In this framework, primary care physicians should provide the most appropriate care for patients taking into account the context surrounding the various backgrounds of patients and medical personnel [25–27]. Even if general practitioners practice primary care in urban areas, being able to provide primary care suitable for the urban contexts by understanding the characteristics of urban contexts and issues may be important.

### “Urban underserved care”

In this study, compared to non-urban areas, one of the characteristic contexts in urban areas was that it is composed of people with diverse socioeconomic conditions and medical needs. Particularly, one of the urban issues is to provide attention to foreigners of racial minorities; “comprehensive care to minority groups” is a competency. In this aspect, general practitioners should understand their context and provide appropriate care for underserved people, such as racial minorities and homeless people, who have difficulty access urban medical resources [28–30]. This competency has already been highlighted as “urban underserved care” and emphasized urban/inner-city program overview of the AAFP’s [11].

### “Fragmentation of care”

Moreover, in urban areas, unlike non-urban areas with limited medical resources, there are various subdivided medical services that even care providers cannot understand. A general practitioner thought that there are circumstances where patients selectively use these services. By this, the scope of clinical practice of general practitioner is narrowed because of multiple specialists in urban areas [7, 31]. As a result, general practitioners felt that medical information of patient is becoming dispersed and care responsibility is becoming unclear. This concept is called “fragmented care” [32–34]. This is one of the tasks in urban context. Research results showed that the urban general practitioner has the ability to actively participate in the management of patients who received divided care to integrate the care after multiple consultations and to work with stakeholders of divided care in urban area. This is a new viewpoint, which has not been pointed out as a competency of general practitioner in urban area.

Additionally, in urban areas, there is a relationship between providers of medical care and welfare personnel in the community; hence, cooperation and face-to-face relationship have been emphasized, but building such relationships is difficult [27, 28]. Our results indicate that physicians need the ability to properly coordinate and adjust care stakeholders and others in cities susceptible to provide divided care.

### Limitations

Limitations of this study include a bias toward general practitioners early in their careers, i.e., those with 7–15 y of experience post-graduation. In order to clarify the definition of general practitioner, this study focused on participants who have certification which began in 2006 and experience in both urban and non-urban (rural) areas. Therefore, generalizability of our findings is limited as the opinions of general practitioners who have worked in urban areas for a long time are not reflected in the data. While 10 participants were sufficient to achieve theoretical sufficiency for competencies of general practitioners in urban areas, theoretical sufficiency was not confirmed for competencies of general practitioners in non-urban (rural) areas and more themes may have been extracted with more participants. Moreover, our study does not include physicians who are not board-certified family physicians but actually engaged in primary care field. Regarding reflexivity, the opinions of general practitioners in non-urban (rural) areas may not have been fully extracted because the interviewer (the main investigator) is a general practitioner who mainly works in urban areas.

## Conclusion

This study was one of the few studies describing the characteristic competencies of urban general practitioners compared with those in non-urban/rural areas. In this study, we provide new perspectives as competency characteristic to urban general practitioners. In addition to the urban underserved care, the ability to integrate fragmented care and properly coordinate and adjust with various care stakeholders are necessary. In contrast, general practitioners in non-urban/rural area are needed to provide a broad scope of care and effective utilization of limited resources of care. In summary, competencies necessary for general practitioners in urban areas is to understand the urban context and to provide contextual care suitable for urban areas. In the modern era where urban population concentration is progressing and the interest in urban health is rising, not only Japan but also other countries will give certain suggestions for primary care education and practice necessary for urban areas.

## Abbreviations

AAFP: The American Academy of Family Physicians
PGY: Post graduate year
SCAT: Steps for Coding and Theorization
